# Infection control for third-party benefit: lessons from criminal justice

**DOI:** 10.1007/s40592-019-00103-y

**Published:** 2019-12-12

**Authors:** Thomas Douglas

**Affiliations:** Faculty of Philosophy, Oxford Uehiro Centre for Practical Ethics, University of Oxford, Suite 8, Littlegate House, 16-17 St Ebbes Street, Oxford, OX1 1PT United Kingdom

**Keywords:** Public health ethics, Infectious disease, Quarantine, Vaccination, Criminal justice ethics, Preventive detention

## Abstract

This article considers what can be learned regarding the ethical acceptability of intrusive interventions intended to halt the spread of infectious disease (‘Infection Control’ measures) from existing ethical discussion of intrusive interventions used to prevent criminal conduct (‘Crime Control’ measures). The main body of the article identifies and briefly describes six objections that have been advanced against Crime Control, and considers how these might apply to Infection Control. The final section then draws out some more general lessons from the foregoing analysis for the ethical acceptability of different kinds of Infection Control.

## Introduction

The question that motivates this article is this: when is it justified to submit an individual to Infection Control for the purposes of protecting third-parties?

By ‘Infection Control’, I mean to pick out interventions that are used to halt the spread of infectious disease and that are somewhat intrusive, in the sense that they would ordinarily infringe (justifiably or unjustifiably) a person’s moral rights; a person subjected to such an intervention would ordinarily have a legitimate moral complaint against the intervener.

Examples of Infection Control would, I think, include quarantine and isolation;^1^ mandatory or strongly incentivised vaccination (for example, vaccination which is required to avoid a large fine, a burdensome administrative procedure, or exclusion of one’s child from a state primary school); and mandatory or strongly incentivised treatment (for example, in the context of mass drug administration intended to eliminate a particular infectious disease from an area).^2^

Though Infection Control interventions would, by definition, ordinarily infringe the targeted person’s rights, I do not claim that they always do so; that person may, for example, have waived the rights that these interventions would otherwise infringe. Moreover, in suggesting that an intervention would ordinarily infringe the target’s moral rights, I do not mean to suggest that it would ordinarily be morally wrong: rights infringements are, I take it, often morally justified, all things considered.

There is, then, an open question regarding whether and when Infection Control is morally permissible, and it is toward this question that the present article is oriented. I will not attempt to arrive at an *answer* to this question. What I will attempt to do is to explore what can be learned that is of relevance to answering it from current thinking about another area in which intrusive interventions are sometimes used for third-party benefit: criminal justice. Our criminal justice systems frequently impose intrusive interventions on criminal offenders, or criminal suspects, at least in part for the purposes of preventing further crime and thereby protecting third parties. I will collectively refer to these interventions as Crime Control. Examples of Crime Control would include pre-trial preventive detention, post-sentence preventive detention, probation regimens, mandatory or strongly incentivised psychological rehabilitation programmes (such as anger management courses and cognitive behavioural therapy), and mandatory or strongly incentivised pharmacological interventions (such as testosterone suppression—so-called ‘chemical castration’—and methadone maintenance therapy).

There is a significant and rapidly growing literature on the ethics of Crime Control, so it seems potentially fruitful to consider what might be learned from that literature for the ethics of Infection Control, especially since the literature on Infection Control remains less well-developed.

There are, of course, important disanalogies between Crime Control and Infection Control. One important disanalogy is that Crime Control interventions are usually—though not always—imposed on individuals who have committed significant crimes. Some might hold that that these individuals *deserve* or have rendered themselves *liable* to certain burdens or constraints. For example, some might hold that, in committing a serious crime, one waives one’s right to free movement and thus renders oneself liable to at least some forms of incarceration. By contrast, Infection Control interventions are normally imposed on individuals who have done nothing that would plausibly render them deserving of, or liable to, significant burdens or constraints.

However, despite this disanalogy, I think we might expect to learn something of relevance for Infection Control interventions from discussions of Crime Control.

One reason for this is simply that, notwithstanding the disanology in question, Crime Control and Infection Control are still, in many important respects, similar. For example, as mentioned above, both are intrusive, and both are imposed in some individuals primarily for the purpose of preventing harms to others.

Another reason is that Crime Control interventions are *sometimes*, like Infection Control interventions, imposed on individuals who have committed no crime; this is most frequently true of interventions, like pre-trial detention, that are imposed prior to conviction. The disanalogy I have mentioned thus does not hold universally.

Finally, yet another reason to pursue the analogy is that much for the discussion of Crime Control has focused on ethical *objections* to it, and the disanalogy in question here does nothing to undermine the thought that these objections might apply to Infection Control as well: after all, if the individuals targeted by Crime Control are deserving of or liable to intrusive interventions, whereas the targets of Infection Control are not, then Crime Control will generally be *less objectionable* than Infection Control. One might think that Infection Control’s targeting of innocent individuals makes it *especially* problematic.

My aim in this chapter is, then, to examine whether and when the most frequently advanced objections to Crime Control would also apply to Infection Control. Each of the following six sections sets out one objection to Crime Control and comments on whether and when a similar objection might apply to some kinds of Infection Control. The final section then seeks to draw some more general lessons from this survey for the ethics of Infection Control, focusing especially on identifying circumstances in which Infection Control may be able to evade the aforementioned objections.

Before proceeding to the main discussion, however, I need to offer three preliminary remarks.

First, I will not, in this chapter, attempt to assess whether the objections that I consider constitute good—let alone decisive—objections to Crime Control. I simply take them as they are, and apply them to Infection Control.

Second, I assume, in the interests of charity, that the objections that I consider are objections to the practice of both (i) subjecting individuals to risk-assessments intended to ascertain the likelihood that they will commit crimes, and (ii) subjecting them to Crime Control interventions on the basis of those risk assessments. Some of the objections that I consider are sometimes presented not as objections to Crime Control itself, but as objections to the risk assessments on which it is based, but I think all of these objections are *most* persuasive when directed at the conjunction of risk-assessment and risk-assessment-based intervention, hence my focus on that conjunctive practice.

Third, to keep the scope of the article manageable, I will consider only objections that might be raised against both physically invasive and physically non-invasive Crime Control interventions. There has been some ethical discussion recently of the possible use of physically invasive medical interventions—such as the injection of testosterone-suppressing drugs—in the prevention of crime (see, e.g., Birks and Douglas 2018). I will not consider the objections that have been raised to such interventions except where they would also plausibly apply to at least some widely used physically non-invasive forms of Crime Control, such as preventive detention.

With these qualifications in hand, let us now turn to the six objections to Crime Control.

## Objection 1: counterproductivity

The first objection that I wish to consider is what I will call the objection from counterproductivity. This objection maintains that at least some forms of Crime Control are morally objectionable because they are in fact counterproductive: they increase the risk that the targeted individual will re-offend. Dawinder Sidhu (2015) advances this worry in relation to criminal sentences whose severity is determined in part by risk-assessment scores. One worry about such sentences, he suggests, is that

‘failure to reform—arguably facilitated by sentences supported by risk-assessment tools—would be a self-fulfilling prophecy…. The sentencer not only actively impairs the offender’s prospects for development by choosing to detain him or her, but then neglects his or her development because the offender is too risky and thus “not worth it”’ (Sidhu 2015, pp. 715–716).

Similarly, writing on the possible use of biological interventions to prevent crime, Horstkötter and collaborators worry that

‘biomedically informed means of early crime prevention could … have negative side effects … [C]hildren might learn to behave as the passive victims of their brains or genes. Moreover, children could experience negative effects on the development of their personal identity, and also parents as well as significant other third persons could come to perceive identified children in a more negative way than perhaps they otherwise would have done. Insofar as this leads to a situation where children are rendered to perceive themselves as “born criminals,” or even adapt their behavior according to their diagnosis, the result could be a self-fulfilling prophecy’ (Horstkötter et al. 2012, p. 27).

We can distinguish three sorts of mechanism via which Crime Control might increase a person’s risk of recidivism.

First, there are *psychological* mechanisms. Individuals deemed to be at high risk of recidivism, and subjected to special interventions on that basis, may come to identify with the ‘dangerous’ label that these assessments and interventions are often taken to imply, and this identification may diminish their motivation to resist pressures towards further crime, perhaps by diminishing their perceived responsibility for their behaviour.^3^

Second, there are *social* mechanisms. Where Crime Control involves bringing offenders together—for example, in prisons—it may enable the development of ‘learned criminality’, as offenders acquire values and skills from one another (Vieraitis et al. 2007, pp. 591, 593). Where it disrupts the offender’s familial and other social relationships—again, as with imprisonment—it may remove social factors that protect against crime (Vieraitis et al. 2007, pp. 590, 592–593, 614–615).

Third, there are *institutional* factors, having to do with the way the ‘high risk’ offender is regarded and treated by criminal justice authorities. Even where risk assessments are originally used to justify additional rehabilitation programmes, the ‘high risk’ label may—as Sidhu suggests in the quote above—ultimately lead authorities to ‘give up’ on these offenders, coming to see them as beyond rehabilitation, and this may increase recidivism by—among other mechanisms—resulting in these offenders being *excluded* from effective rehabilitation programmes.

Might an analogue of the counterproductivity objection also be raised against Infection Control measures, such as quarantine or compulsory vaccination? This might seem initially implausible; the mechanisms—just described—via which Crime Control is thought to have counterproductive effects look to be rather particular to Crime Control, or at least to criminal justice contexts.

However, the counterproductivity objection to Crime Control does invite us to consider whether there are different mechanisms via which Infection Control would be counterproductive—that is, would increase infectious risk. Two possibilities suggest themselves.

An initial possibility is that Infection Control might undermine trust in healthcare. In some cases, Infection Control is implemented by healthcare professionals but differs from ordinary forms of healthcare in being motivated substantially by the goal of protecting third parties, rather than purely by the goal of advancing the target’s own best interests. One might worry that, in breaking the link between the healthcare professions and the advancement of the patient’s own interests, such interventions will weaken trust in the healthcare professions, perhaps in a way that might lead to worse healthcare outcomes, for example, because those subjected to Infection Control will be less likely to seek out healthcare in the future.

Second, in some cases Infection Control might contribute to increased infectious risk through biological mechanisms. For example, when individuals deemed at risk of being infected with a pathogen are quarantined along with other ‘high risk’ individuals, those who are in fact uninfected may become infected as a result.

Given these possibilities, it seems plausible that an analogue of the counterproductivity objection might apply to some forms of Infection Control, though the mechanisms underlying the counterproductivity are likely to differ between criminal justice and public health contexts.

## Objection 2: stigmatisation

A second concern often raised regarding Crime Control measures is that they—or the assessments of high forensic risk on which they are based—are stigmatising. Criminal offenders in general are frequently the objects of significant disapproval, which may be intrinsically harmful and lead to many other forms of disadvantage, and that disapproval may be stronger for those deemed to pose an especially high risk (von Hirsch 1972, p. 743; Silver and Miller 2002; Horstkötter et al. 2014). Moreover, there is the possibility that stigmatisation of high risk offenders will generalise to others who have not committed crimes—and may not be at high risk of doing so—but who share one or more of the risk factors deployed by the risk assessment tool, or otherwise resemble individuals assessed as high risk (Silver and Miller 2002). For example, if offenders deemed to be high risk are disproportionately afflicted by mental health conditions—and especially if diagnosed mental health conditions are used as an explicit predictor of recidivism in risk assessment tools—we might worry that others with those diagnoses will be stigmatised as potential criminals.

It seems to me that Infection Control—and the assessments of high *infectious* risk on which it is based—could be similarly stigmatising. After all, like criminality, many infectious diseases have significant negative associations in the public imagination, with HIV being the most obvious example (e.g., Van Brakel 2006; Whittle et al. 2017; Dubov et al. 2018; Chambers et al. 2015).

It might be argued that the negative stigma associated with declarations of high forensic risk is different in kind from that associated with declarations of high infectious risk. The former typically has a moral character, whereas the latter typically does not. Those declared to pose a high forensic risk are not merely stigmatised as dangerous, they are also deemed to be morally flawed, perhaps in part because the Crime Control interventions that tend to follow such judgments—such as preventive detention—are sometimes taken to communicate an official expression of moral condemnation and not to be purely preventive measures. It could be argued that, other things being equal, it is worse to be subjected to the kind of moral stigma associated with crime than to other forms of stigma.

However, the posited difference in the nature of the stigma is not universal. Some infectious diseases are associated with heavily moralised behaviours such as illicit drug use and homosexual or unprotected sex, and these diseases frequently carry a stigma that is moral in nature (Chambers et al. 2015; Frank and Nagel 2017). Quarantine measures may help to reinforce that stigma.

Moreover, one might think that when the stigma associated with infectious diseases *is* moralised, this moralisation will be more problematic than in the case of stigma associated with forensic risk; it might be argued that it is appropriate that individuals who have committed serious crimes are subjected to *some* moral disapproval; it is difficult to see how a moral stigma could be appropriate in typical cases of infectious disease.

## Objection 3: inequality

A third commonly advanced objection to Crime Control adverts to its tendency to exacerbate socio-economic inequalities. To the extent that (i) assessed forensic risk tracks socio-economic disadvantage, and (ii) Crime Control interventions imposed on those deemed high risk themselves contribute to socio-economic disadvantage, these interventions will ratchet up existing inequalities (Sidhu 2015; Gavaghan et al. 2014, p. 25). It can plausibly be argued that both (i) and (ii) hold: disadvantaged groups *are* overrepresented among those deemed to be high risk by commonly used forensic risk assessment tools, and it is plausible that both the longer periods of incarceration and the stigmatisation endured by these individuals tend to increase levels of disadvantage (Hannah-Moffat and Struthers Montford 2019). On some views, the burdens and constraints involved in Crime Control in fact partly *constitute* socio-economic disadvantage: part of what it is to be disadvantaged is to lack basic freedoms and opportunities afforded to others.^4^

Similar concerns could be raised regarding the inegalitarian effects of Infection Control. Because these interventions—or the infectious risk assessments on which they are based—can lead to both negative stigma and an increased risk of becoming infected, they can contribute to disadvantage. Indeed, some forms of Infection Control—like quarantine—also involve constraints on basic freedoms and opportunities that are arguably part of what it is to be disadvantaged. Moreover, in some cases the assignment of high infectious disease risk can be expected to track pre-existing disadvantage, because acquisition of some infectious diseases is correlated with prior disadvantage. For example, within Europe alone, there is evidence that meningococcal meningitis, hepatitis A and Methicillin-resistant Staphylococcus aureus are associated with low socio-economic status (Twisselmann 2000; Bagger et al. 2004; Williams et al. 2004; Hrivniaková et al. 2009), while tuberculosis disproportionately afflicts persons from a range of frequently disadvantaged populations including immigrants, homeless people, users of illicit substances, prisoners, and HIV positive persons (Semenza and Giesecke 2008; Klinkenberg et al. 2009; ECDC and WHO Europe 2013). Where Infection Control measures are targeted at these groups, they are likely to result in an exacerbation of socio-economic inequality.

## Objection 4: disproportionality

Perhaps the most frequently advanced objection to Crime Control holds that it flaunts the requirement that the institutions of criminal justice not impose heavier burdens or stricter constraints than an individual deserves, or, as I take to be equivalent, that they not impose burdens or constraints that are disproportionately severe relative to the individual’s culpability (Morse 2011; Gavaghan et al. 2014, p. 75). (This requirement is often called the ‘proportionality constraint’ or the ‘negative retributivist constraint’.) Because decisions about when to cease Crime Control interventions, like preventive detention, are normally based on risk, rather than on a person having ‘served their time’, these interventions can, in individuals whose forensic risk remains high, be imposed for very long periods; offenders subjected to preventive detention may be detained for much longer than they would have been incarcerated in a purely retributive regime and, most importantly for our purposes here, for longer than proportionality requires. Crime Control interventions can also, of course, transgress the proportionality constraint because they are imposed on individuals who have in fact committed no moral wrong. This is commonly the case for interventions, like pre-trial detention, that are imposed on criminal suspects rather than convicted offenders. It will also be the case for Crime Control interventions imposed in relation to crimes that are not in fact moral wrongs, like the past crime of sodomy.

Concerns such as these are not commonly raised in relation to Infection Control. True, the concept of proportionality does often come up in public health ethics (see, e.g., Childress et al. 2002, p. 173), but the understanding of proportionality is normally rather different from that employed in forensic contexts: in relation to Infection Control—and public health more generally—the claim is typically that burdens and constraints imposed on individuals must be proportionate to the magnitude of the harm that is (expectably) averted by those interventions; proportionality *to culpability* does not come into the picture.^5^

Still, it seems that concerns about proportionality-to-culpability *could* also be raised regarding Infection Control. Infection Control can impose severe burdens and constraints, and these are typically imposed on individuals who are culpable for no wrong—or at least, do not bear a degree of culpability that might plausibly render them deserving of the severe constraints and burdens that are imposed. It might thus seem that Infection Control, like pre-trial detention imposed on individuals who will turn out to be innocent, *routinely* violates proportionality-to-culpability.^6^

It is true that some individuals subjected to Infection Control are somewhat culpable for the infectious risk that they pose to others. Consider cases in which people pose an infectious risk to others by virtue of carrying a disease that was acquired through unsafe sexual practices or as a result of refusal to receive a safe and effective vaccine. These people, we might think, bear some culpability. But even in these cases, the overall degree of culpability will typically be low, and surely not sufficient to render significant burdens and constraints—like those involved in quarantine—proportionate.

## Objection 5: discrimination

A worry about risk-based Crime Control that has recently come to prominence holds that it is discriminatory because it subjects some to less favourable treatment than others (partly) on the basis of group-based predictors of risk.

Sidhu (2015, p. 675) puts the concern thus:

‘risk-assessment tools identify risk on the basis of an offender’s group membership or group identity, assign the same monolithic risk profile to everyone in the group, and premise punishment on group characteristics which the individual possesses by accident of birth or cannot otherwise meaningfully change’.

There is, of course, disagreement on when and why it is wrongfully discriminatory (henceforth just ‘discriminatory’) to employ group-based variables as bases for disfavourable treatment. Sidhu’s thought seems to be that it is discriminatory when and because the group-based variables are not within the control of the members of the group (see, for a similar view, Kahlenberg 1996, pp. 54–55). Other views would hold that it is—or can be—discriminatory only when and because the group is a socially salient one (like an ethnic or gender group, and unlike the group of people who are more than 1.85 metres tall) (e.g., Lippert-Rasmussen 2013, Ch. 1), the group has previously been a target of group-based oppression (as is the case for women, and many ethnic minority groups) (e.g., Scanlon 2008, p. 247), or the group is defined in a way that is coarser than necessary to pick out risky individuals, so that many group members are unnecessarily labelled as ‘high risk’ (e.g., Thomas 1992).

There is also disagreement about whether discrimination requires *explicit* use of group-based variables as predictors. This issue recently came to the fore in a controversy over the COMPAS risk assessment algorithm, which is widely used in the US criminal justice system (Angwin et al. 2016; Dieterich et al. 2016; Angwin and Larson 2016; Corbett-Davies et al. 2016). This algorithm does not use race as an explicit predictor of risk, but it does tend to assign higher risk scores to African American men than to White American men, and also has higher false positive rates in African American men than in White American men; Black American men are more likely to be *wrongly* labelled as ‘high risk’ than White American men. This led some to hold that it is racially biased in a morally objectionable way (see, e.g., Angwin et al. 2016).

Finally, there is also disagreement about why, precisely, discrimination is wrong (see Lippert-Rasmussen 2013, Part II). There is thus scope for disagreement about the precise nature of the moral objection being advanced by those who hold that risk-based Crime Control is discriminatory. There are, I think, three plausible views.^7^

On the first view, the objection is closely related to that described in the previous section; the worry is that risk-based Crime Control tends to disrupt a desert-based distribution of goods and ills.^8^ This view meshes well with the account of discrimination according to which it consists in disfavourable treatment based on group-based variables *that are not within the control of the group members*; we may be concerned about the lack of control because we take it to indicate that the individual is not culpable for their membership of that group, and thus that there is no reason to think that the disfavourable treatment will be proportionate to culpability.

On the second view, the objection is closely related to that set out in the section ‘Objection 3: Inequality’; the worry is that risk-based Crime Control may exacerbate the disadvantage already endured by the discriminated-against group. This view meshes well with the account of discrimination according to which discrimination consists in disfavourable treatment based on group-based variables *that are markers of previous oppression*; we may worry that the disfavourable treatment will deepen the disadvantages that have already been brought about by that oppression.

Finally, on the third view, the objection is that risk-based Crime Control involves gratuitously harming people by imposing burdens and constraints on a needlessly large group of people. This view meshes well with the account of discrimination according to which it consists in treating people disfavourably on the basis of *overly coarse* group-based variables.

Might these three variants of the discrimination objection apply also to Infection Control? I have already argued that Infection Control might often impose burdens and constraints that are not proportionate to culpability, and that exacerbate existing disadvantage, so it seems clear that the first two variants of the objection could well apply. It is also, I think, clear that they could involve wrongs of the third type. Consider a case in which quarantine is imposed on everyone who lives in a particular neighbourhood where an infectious disease has been spreading, and suppose that the quarantine would have been just as effective—and in no other respect worse—had it been imposed only on a subset of these people—say, those who live in this neighbourhood *and* are known to have had contact with an infected individual. Clearly, in this case, the authorities gratuitously harm all residents of the neighbourhood who have not had known contact with an infected individual.^9^

## Objection 6: respect

A sixth objection to at least some forms of Crime Control holds that they are *disrespectful* by virtue of failing to treat those whom they target as rational agents. The objection has perhaps been most forcefully and frequently advanced in relation to preventive detention, which, according to Andrew von Hirsch, treats the detained individuals as if they were ‘beasts in a circus … beings that must be restrained, intimidated, or conditioned into submission because they are incapable of understanding that harmful conduct is wrong’ (von Hirsch 1992, p. 67). In a similar vein, Barbara Underwood (1979, p. 1414) holds that ‘[t]o imprison a person because of the crimes he is expected to commit denies him the opportunity to choose to avoid those crimes…[R]espect for individual autonomy requires recognition of the possibility that an individual can choose to refute any prediction about himself.’

The respect objection is not limited to preventive detention, however; it could plausibly be advanced against all forms of Crime Control that do not engage the rational capacities of the targeted individual(s). It has, for example, also been advanced against the use of pharmaceutical interventions—such as chemical castration—to promote rehabilitation. Elizabeth Shaw holds that some such interventions ‘could be viewed as objectification, treating the individual merely as a means, or failing to respect the individual’s rational agency’ (Shaw 2018a, p. 3; see also Shaw 2018b, p. 268; Bublitz and Merkel 2014, p. 73; Bublitz 2016, p. 99).

Could the respect objection also be advanced against Infection Control? I am not aware of it having been so-advanced. I suspect this may have to do with the fact that, unlike forensic risks, infectious risks are not generally thought of as risks of what we might call *rational harms*—harms that are largely the product of the rational agency (e.g., choices) of the ‘risky individual’. Crime Control interventions seek to prevent harms that arise (in most cases relatively directly) from the choices of the criminal offender, whereas Infection Control interventions arguably seek to prevent harms in which the choices of the infected individual play no important causative role. Perhaps the thought is that we are only required to employ rationality-engaging risk mitigation strategies when the risk is itself a risk of rational harm. After all, we might think it is only in these cases that rationality-engaging interventions could be effective in mitigating the harm.

There are, however, some cases in which the harms that Infection Control seeks to prevent are, to a significant degree, attributable to the choices of the infected individual. Consider cases in which a person has acquired a condition through unsafe sexual practices and may cause harm to others through continuing to engage in such practices. Or consider a case in which a person will continue to pose an infectious risk to others only if she chooses not to avail herself of an available treatment. It seems plausible to think that those who advance the respect objection to Crime Control should also require (or at least prefer) rationality-engaging means of risk-reduction in cases such as these. Perhaps, for example, we ought to mitigate the risk posed by the sexual risk-taker by *convincing* him to practice safe sex.

## Lessons

I have outlined six objections that are sometimes raised to Crime Control and suggested that each plausibly applies—at least in some ways and in some cases—to Infection Control as well.

In this final section, I seek to draw some more positive lessons from the foregoing discussion. What can we learn from it for the ethics of Infection Control? Each of the lessons takes the form of an observation regarding when Infection Control may be less morally problematic than otherwise because it evades one or more of the objections that I have raised.

First, I think my discussion suggests that Infection Control measures will normally be less problematic when prior attempts have been made to mitigate infectious risk through encouraging an individual to submit to voluntary risk-reduction measures than when no such attempts have been made. When encouragement of voluntary measures has been tried, but has failed, this may constitute evidence that risk can no longer be reduced through rationality-engaging means, so that the requirement to pursue such means no longer applies. Moreover, attempting voluntary interventions first may eliminate or weaken proportionality-based objections to Infection Control, since one might think that a person can become culpable for the risk that she poses through refusing voluntary interventions, at least if those interventions pose minimal risks to that person. If, through refusing voluntary interventions, a person becomes somewhat culpable for the infectious risks that she poses, this will—other things being equal—weaken the proportionality-based objections to imposing Infection Control measures, since *some* burdens or constraints will now count as proportionate.

Second, proportionality-based objections will, of course, *already*, even prior to attempts at voluntary measures, be weaker in certain cases: those in which the infectious risk was acquired through culpable wrongdoing on the part of the ‘risky’ individual. However, the practical relevance of this point is perhaps limited, since those implementing Infection Control will generally not be in a position to determine whether and to what extent a person’s risk is the result of culpable negligence. Also, even if Infection Control is less problematic with respect to proportionality when the infectious risk was acquired through culpable wrongdoing than when it was not, it may in other respects be more problematic in such cases. For example, we might worry that stigmatisation will typically be a greater problem in these cases than others, since the stigma is more likely to be moralised.

Third, one obvious lesson that can be drawn from the discussion of inequality is that Infection Control will generally be less concerning in cases where the infectious disease being targeted is not one that tracks prior disadvantage.

Similarly, fourth, the discussion of stigma suggests that Infection Control will be less problematic where the targeted infectious disease is not associated with stigmatised behaviours (like illicit drug use or homosexual sex) or stigmatised groups (often ethnic minorities). Fifth, Infection Control will be less problematic with respect to counterproductivity when the infection control intervention does not itself expose the targeted individuals to greater risk of infection. And sixth, it will be less problematic when the assessment of infectious risk is fine grained—not, for example, based on possession of a small number of coarse demographic factors like sex, age and city of residence.

Finally, seventh, Infection Control will generally be less problematic when the constraints and burdens that it imposes can and will be compensated. For example, quarantine will be less problematic when quarantined individuals are compensated for inconvenience, disruption and lost earnings during the period of quarantine. Such compensation will weaken at least two of the objections mentioned above—those adverting to inequality and disproportionality—since these objections rely on the thought that Infection Control is harmful for those subjected to it, and compensation could diminish its harmfulness.

## Footnotes

1 ‘Isolation’ standardly refers to the separation from others of individuals infected with an infectious agent, while ‘quarantine’ refers to the separation of individuals merely at risk of being infected.

2 I do not consider the differences between mandatory and strong incentivized interventions in this article, but many of the concerns that I will raise regarding Infection Control would plausibly be more serious in relation to mandatory interventions.

3 For example, Horstkötter and de Wert worry that ‘emphasizing a child’s risk status might negatively affect his/her self-perception and identity development and lead to the abdication of personal responsibility’ (Horstkötter and de Wert 2013, p. 20; see also Horstkötter et al. 2014, pp. 71, 76).

4 This is so, for example, on John Rawls’ theory of justice, which uses endowment of ‘primary social goods’ as the metric of socio-economic disadvantage, and takes basic political freedoms and social opportunities as among those goods (Rawls 1999, pp. 54, 79, Ch. IV).

5 There is, it seems to me, an interesting question regarding whether the kind of proportionality typically considered in Infection Control contexts (i.e., proportionality-to-harm-averted) ought to be given greater consideration also in relation to Crime Control. However, since I am concerned with inferences in the other direction here, I do not consider this matter further.

6 It might be argued that the institutions of public health are not bound by the same desert-based moral constraints as those of criminal justice. That is, it might be argued that public health institutions fall under no analogue of the proportionality requirement. Thus, even if both Crime Control and Infection Control impose undeserved burdens and constraints, this may violate a moral requirement in the case of preventive detention, but not in that of quarantine. I am, however, aware of no good argument for the view that a proportionality constraint applies in criminal justice but not in public health (see, for a fuller discussion of this issue, Douglas 2019).

7 There are other explanations for why different kinds of discrimination are wrongful (see, for discussion, Lippert-Rasmussen 2013, Part II). I focus here on explanations for the wrongfulness of discrimination that plausibly apply to the case of risk-based Crime Control measures.

8 For versions of this worry applied to discrimination in general, see, for example, Arneson (1999, pp. 239–240) and Lippert-Rasmussen (2013, pp. 165–183).

9 In 1900, the Circuit Court of the Northern District of California appeared to have been moved by considerations of this sort. It found quarantine arrangements employed to prevent a possible outbreak of bubonic plague in San Francisco to be unreasonable, partly on the basis that they applied to an over-broad group: the over 10,000 residents of a particular area. (The court also deemed that the arrangements were discriminatory on the basis that they were selectively enforced against Chinese people.) See Jew Ho v. Williamson, 103 F. 10 (N.D. Cal. 1900). I thank an anonymous reviewer for drawing this case to my attention.
